# Seasonal dynamics of ammonia/ammonium-oxidizing prokaryotes in oxic and anoxic wetland sediments of subtropical coastal mangrove

**DOI:** 10.1007/s00253-012-4510-5

**Published:** 2012-10-26

**Authors:** Yong-Feng Wang, Yao-Yu Feng, Xiaojun Ma, Ji-Dong Gu

**Affiliations:** 1 Laboratory of Environmental Microbiology and Toxicology, School of Biological Sciences, The University of Hong Kong, Pokfulam Road, Hong Kong, SAR People’s Republic of China; 2 State Environmental Protection Key Laboratory of Environmental Risk Assessment and Control on Chemical Process, School of Resources and Environmental Engineering, East China University of Science and Technology, Shanghai, 200237 People’s Republic of China; 3 School of Life Sciences, Lanzhou University, Lanzhou, 730000 People’s Republic of China; 4 The Swire Institute of Marine Science, The University of Hong Kong, Shek O, Cape d’Aguilar, Hong Kong, SAR People’s Republic of China

**Keywords:** Anammox bacteria, AOA, AOB, *AmoA*, Mangrove, Sediment type, Seasonal dynamics

## Abstract

Mangrove wetlands are an important ecosystem in tropical and subtropical regions, and the sediments may contain both oxic and anoxic zones. In this study, ammonia/ammonium-oxidizing prokaryotes (AOPs) in yellow and black sediments with vegetation and non-vegetated sediments in a mangrove wetland of subtropical Hong Kong were investigated in winter and summer. The phylogenetic diversity of anammox bacterial 16S rRNA genes and archaeal and bacterial *amoA* genes (encoding ammonia monooxygenase alpha-subunit) were analyzed using PCR amplification and denaturing gradient gel electrophoresis to reveal their community structures. Quantitative PCR was also used to detect their gene abundances. The results showed that seasonality had little effect, but sediment type had a noticeable influence on the community structures and abundances of anammox bacteria. For ammonia-oxidizing archaea (AOA), seasonality had a small effect on their community structures, but a significant effect on their abundances: AOA *amoA* genes were significantly higher in winter than in summer. In winter, the vegetated yellow sediments had lower AOA *amoA* genes than the other types of sediments, but in summer, the vegetated yellow sediments had higher AOA *amoA* genes than the other types of sediments. Sediment type had no apparent effect on AOA community structures in winter. In summer, however, the vegetated yellow sediments showed obviously different AOA community structures from the other types of sediments. For ammonia-oxidizing bacteria (AOB), seasonality had a significant effect on their community structures and abundances: AOB *amoA* genes in winter were apparently higher than in summer, and AOB community structures were different between winter and summer. Sediment type had little effect on AOB community structures, but had a noticeable effect on the abundances: AOB *amoA* genes of the vegetated yellow sediments were obviously lower than the black ones in both seasons. This study has demonstrated that seasonality and sediment type affected community structures and abundances of AOPs differently in oxic and anoxic sediments of the mangrove wetland.

## Introduction

Ammonia/ammonium-oxidizing prokaryotes (AOPs), including anaerobic ammonium-oxidizing (anammox) bacteria and aerobic ammonia-oxidizing archaea (AOA) and bacteria (AOB), are three groups of microorganisms that are responsible for ammonium/ammonia oxidation in the global nitrogen cycle. Anammox bacteria transform ammonium, together with nitrite, into dinitrogen gas (N_2_) under anaerobic conditions (Mulder et al. 1995). Five genera of anammox bacteria have been recognized, and all of them are within the phylum Planctomycetales: *Candidatus* Brocadia (Strous et al. 1999), *Ca*. Kuenenia (Schmid et al. 2000), *Ca*. Scalindua (Schmid et al. 2003), *Ca*. Anammoxoglobus (Kartal et al. 2007), and *Ca*. Jettenia (Quan et al. 2008). In addition to their application in inorganic N removal of sewage treatment (Kartal et al. 2010), anammox bacteria have been demonstrated to exist in marine environments, including marine water column and sediments (Dang et al. 2010; Hong et al. 2011; Kuypers et al. 2003, 2005; Lam et al. 2009), coastal and estuary sediments (Dale et al. 2009; Li et al. 2011c), coastal mangrove wetlands (Cao et al. 2011a; Li et al. 2011b, c), and even polar marine sediments and sea ice (Rysgaard and Glud 2004). It was estimated that anammox bacteria might be responsible for more than 50 % of global nitrogen losses from the oceans (Brandes et al. 2007; Kuypers et al. 2003). In addition to marine environments, anammox bacteria were also detected in freshwater and terrestrial environments such as lakes (Hamersley et al. 2009; Schubert et al. 2006), rivers (Zhang et al. 2007), terrestrial soils (Penton et al. 2006), rice paddy fields (Wang and Gu 2012a), groundwater (Clark et al. 2008), hot springs (Jaeschke et al. 2009), and even oil fields (Li et al. 2010a). Functional genes encoding hydrazine oxidoreductase (*hzo*), nitrite reductase (*nir*), and hydrazine synthase (*hzs*) have been used to detect anammox bacteria in natural environments (Harhangi et al. 2012; Li et al. 2010b; Schmid et al. 2008), but the more widely used molecular biomarker remains to be the 16S rRNA gene, currently.

AOA and AOB are able to oxidize ammonia with oxygen to nitrite, which is the first and rate-limiting step in nitrification (Purkhold et al. 2000). AOB were identified as early as more than one century ago (Winogradsky 1890), but AOA were discovered more recently (Könneke et al. 2005). To date, all known AOB fall into two phylogenetic lineages within β- and γ-Proteobacteria (reviewed by Kowalchuk and Stephen 2001). Based on genomic level comparison, AOA was classified into the newly proposed phylum Thaumarchaeota (Brochier-Armanet et al. 2008; Pester et al. 2011). Although AOB and AOA are affiliated with different domains, both of them contain homologous ammonia monooxygenase (AMO), which oxidizes ammonia with oxygen to hydroxylamine. The gene *amoA*, encoding the alpha subunit of AMO, has been widely used as a functional gene marker to analyze the phylogeny and abundance of AOB and AOA in natural environments (Francis et al. 2005; Rotthauwe et al. 1997).

Denaturing gradient gel electrophoresis (DGGE) is an effective approach to analyzing the genetic diversity of environmental microbial populations by separating PCR amplicons of target genes of equal lengths in polyacrylamide gels containing a linearly increasing gradient of denaturants (Muyzer et al. 1993). Compared with traditional methods of establishing clone libraries, DGGE can provide direct information on community structure changes and, more importantly, is a time- and cost-effective approach when a considerable number of samples are analyzed. Because of this advantage, the DGGE technique has been widely utilized to analyze different microbial population dynamics, including AOA (Hussain et al. 2011; Park et al. 2008) and AOB (Cébron et al. 2004; Nicolaisen and Ramsing 2002; Tourna et al. 2010).

Mangrove wetlands, mainly distributed in the tropics and subtropics, are an intertidal ecosystem between terrestrial and marine ecosystem and harbor unique microbial functional groups and diversity (Nagelkerken et al. 2008). Mangrove ecosystem supports abundant life and possesses high diversity (Smith et al. 1991). Plants are the most important primary producers in wetlands. In addition to being highly productive, plants affect microorganisms in wetlands by transferring oxygen to the vicinity of roots, the rhizosphere (Zhang et al. 2009), and exude various substances to act as a food source for the microorganisms surviving in the habitat (Lu et al. 2007). Because of the activity of roots and associated microorganisms, the sediments in the mangrove wetlands could be easily classified into two types by colors: yellow sediment and black sediment. The vegetated yellow sediments are in the vicinity of the roots and are significantly affected by the activity of the roots and the associated microorganisms. The vegetated black sediments lack roots; therefore, the effects of plant roots can be minimum. But both of them are under the direct influence of mangrove leaf litters. Besides the vegetated yellow and black sediments, non-vegetated sediments located out of the mangrove wetland were also affected by the mangroves to a certain degree as the water and litters in the mangrove wetland could be transported out by tidal activity (Kristensen et al. 2008).

AOPs in some wetlands have been investigated (Dale et al. 2009; Herrmann et al. 2008, 2009). In particular, a number of studies had been carried out on AOP communities in the mangrove wetland at Mai Po Nature Reserve in Hong Kong (Cao et al. 2011a, b, 2012; Li et al. 2011a, c). However, these studies mainly focused on the AOPs in the non-vegetated mudflat and estimated the influences of anthropogenic pollution. We investigated AOP communities in the rhizosphere and the non-vegetated sediments in a young mangrove wetland, which showed that the roots of young mangrove trees had little influence on the community structures of AOPs (Wang and Gu 2012a, b). In the present study, we investigated AOPs in different types of sediments of the mangrove wetland in Mai Po Nature Reserve in Hong Kong. In particular, DGGE of 16S rRNA genes of anammox bacteria and *amoA* genes of AOA and AOB was employed to analyze their community structures in different types of sediments in winter and summer.

## Materials and methods

### Description of sampling sites and strategies

Sediment samples were collected from the mangrove wetland in Mai Po Nature Reserve of Hong Kong, China, which was designated as a Ramsar site in 1995. It includes freshwater ponds, traditional shrimp ponds, reedbeds, an intertidal mudflat, and a mangrove wetland. The dominant plants in the mangrove wetland are *Kandelia obovata*. Further description of the location is available elsewhere (Li et al. 2011c).

Sediment samples were collected from three different sites in the mangrove wetland (22°29′30″ N, 114°2′2″ E; 22°29′33″ N, 114°2′6″ E; and 22°29′45″ N, 114°1′59″ E) in winter (December 21, 2010) and summer (June 25, 2011). The average daily temperatures were 20.3 and 29.8 °C for winter and summer, respectively (data calculated from http://www.hko.gov.hk/wxinfo/pastwx/past.htm). At each site, the vegetated yellow and black sediments and the non-vegetated sediments 10 m away from the mangrove wetland were collected at depth of 0–15 cm. Each sample was a composite of four portions of 500 g sediment after mixing. A total of 18 composite samples were collected. All sediment samples were immediately put into individual plastic bags and ice boxes for transporting back to the laboratory after collection in the same day. In the laboratory, each sample was mixed thoroughly and 100 g of sediment were kept at −80 °C for subsequent molecular analysis; the remaining sediment for physiochemical analysis was processed immediately.

### Physicochemical analyses

Redox potentials and pH of the sediments were measured in triplicate in situ with the IQ160 pH meter (with ORP electrode; IQ Scientific Instruments, Inc.). Pore water of the sediments was acquired by centrifugation at 4 °C, 5,000 rpm for 20 min using a Hitachi high-speed refrigerated centrifuge CR21F (Japan). Ammonium-N and nitrite-N of the sediment pore water were determined in duplicate with the Lachat QuikChem 8000 Flow Injection Analyzer (Lachat Instruments, Inc.). The measuring procedures were in accordance with the manual of the instrument. Sediment dry weights were measured in triplicate after drying in an oven at 105 °C for 24 h until constant weight was achieved. Organic matter of each sediment was measured in triplicate using loss-on-ignition method (Heiri et al. 2001), in which organic matter was oxidized at 500–550 °C for 2 h in a Thermolyne Muffle Furnace (type 47900).

### Sediment DNA extraction

Total DNA of each sediment sample was extracted in duplicate using the PowerSoil^®^ DNA isolation kit (MO BIO Laboratories, Inc. USA) according to the manual of the manufacturer. The extracted DNA from the same sediment in duplicate was then mixed for subsequent molecular analysis and stored at −20 °C after use.

### PCR amplification

The PCR-amplified products by nested PCR were used for DGGE; the methods had been reported before (Auguet et al. 2011; Nicol et al. 2008; Nicolaisen and Ramsing 2002; Park et al. 2008; Verhamme et al. 2011). Briefly, the genes were first amplified using PCR primers without a GC clamp. The products were then checked by agarose gel electrophoresis and the expected size bands were cut and purified. The purified products were then diluted and used as templates for a second round of PCR using primers with a GC clamp (5′-CCGCCGCGCGGCGGGCGGGGCGGGGGCACGGGG-3′; Muyzer et al. 1993). This method has been demonstrated to be unbiased and effective if the cycles and template quantity were well controlled (unpublished data). The detailed protocols are as follows.

The 16S rRNA gene fragments of anammox bacteria were first amplified using Amx368F (5′-TTCGCAATGCCCGAAAGG-3′) and Amx820R (5′-AAAACCCCTCTACTTAGTGCCC-3′). The optimized PCR mixture contained in a final volume of 50 μl was as follows: 1.5 μl of DNA (20 ng μl^−1^), 10 μl of 5× GoTaq Flexi buffer (Promega, Hong Kong), 4 μl of MgCl_2_ (25 mM, Promega), 1 μl of dNTPs (10 mM of each, Promega), 1 μl of each forward and reverse primers (20 μM), 0.25 μl of GoTaq Flexi polymerase (5 U μl^−1^, Promega), and 5 μl of BSA (0.1 %). PCR conditions were set as follows: 94 °C for 4 min; 30 cycles of 95 °C for 45 s, 59 °C for 50 s, followed by 72 °C for 1 min; and finally 72 °C for 15 min. PCR products were checked by electrophoresis in 1 % agarose gel stained with GelRed™ (Biotium, Hayward) at 1:10,000 and then purified using Gel Advance™ Gel Extraction System (Viogene-Bio Tek Co., Taiwan, ROC). The purified PCR product was then amplified with Amx368F-GC and Amx820R. The PCR mixture contained in a final volume of 50 μl was as follows: 2 μl of DNA (0.1 ng μl^−1^), 10 μl of 5× GoTaq Flexi buffer (Promega), 4 μl of MgCl_2_ (25 mM), 1 μl of dNTPs (10 mM of each, Promega), 1 μl of each forward and reverse primers (20 μM) and 0.25 μl of GoTaq Flexi polymerase (5 U μl^−1^, Promega), and 5 μl of BSA (0.1 %). PCR conditions were set as follows: 94 °C for 4 min; 17 cycles of 95 °C for 45 s, 63 °C for 50 s, followed by 72 °C for 1 min; and finally 72 °C for 7 min. PCR products were checked by electrophoresis in 1 % agarose gel stained with GelRed (1/10,000). The PCR products were then subject for subsequent DGGE analysis.

The archaeal *amoA* genes were first amplified using the primers Arch-*amoA*F (5′-STAATGGTCTGGCTTAGACG-3′) and Arch-*amoA*R (5′-GCGGCCATCCATCTGTATGT-3′). Based on the standard procedures in the manufacturer’s instructions and results of previous studies (Francis et al. 2005), the optimized PCR mixture contained in a final volume of 50 μl was chosen as follows: 1.5 μl of DNA (20 ng μl^−1^), 10 μl of 5× GoTaq Flexi buffer (Promega), 3 μl of MgCl_2_ (25 mM, Promega), 1 μl of dNTPs (10 mM of each, Promega), 1 μl of each forward and reverse primers (20 μM), 0.25 μl of GoTaq Flexi polymerase (5 U μl^−1^, Promega), and 5 μl of BSA (0.1 %). PCR conditions were set as follows: 95 °C for 5 min; 30 cycles of 94 °C for 45 s, 53 °C for 1 min, and 72 °C for 1 min; and finally 72 °C for 15 min. PCR products were checked by electrophoresis in 1 % agarose gel stained with GelRed (1/10,000) and then purified using Gel Advance. The purified PCR product was then amplified with Arch-*amoA*F-GC and Arch-*amoA*R. The optimized PCR mixture contained in a final volume of 50 μl was as follows: 2 μl of DNA (0.1 ng μl^−1^), 10 μl of 5× GoTaq Flexi buffer (Promega), 3 μl of MgCl_2_ (25 mM,), 1 μl of dNTPs (10 mM of each, Promega), 1 μl of each forward and reverse primers (20 μM), 0.25 μl of GoTaq Flexi polymerase (5 U μl^−1^, Promega), and 5 μl of BSA (0.1 %). PCR conditions were set as follows: 95 °C for 5 min; 17 cycles of 94 °C for 45 s, 58 °C for 1 min, followed by 72 °C for 1 min; and finally 72 °C for 15 min. The PCR product was checked by electrophoresis in 1 % agarose gel stained with GelRed (1/10,000). The PCR products were then subject to subsequent DGGE analysis.

The bacterial *amoA* genes were amplified using the primers *amoA*-1F (5′-GGGGGTTTCTACTGGTGGT-3′) and *amoA*-2R (5′-CCCCTCKGSAAAGCCTTCTTC-3′). The optimized PCR mixture contained in a final volume of 50 μl was chosen as follows: 1.5 μl of DNA (20 ng μl^−1^), 10 μl of 5× GoTaq Flexi buffer (Promega), 2.5 μl of MgCl_2_ (25 mM, Promega), 1 μl of dNTPs (10 mM of each, Promega), 1 μl of each forward and reverse primers (20 μM), 0.25 μl of GoTaq Flexi polymerase (5 U μl^−1^, Promega), and 5 μl of BSA (0.1 %). PCR conditions were set as follows: 94 °C for 3 min; 30 cycles of 94 °C for 45 s, 55 °C for 45 s, and 72 °C for 50 s; and finally 72 °C for 10 min. PCR products were checked by electrophoresis in 1 % agarose gel stained with GelRed (1/10,000) and then purified using Gel Advance. The purified PCR product was then amplified with *amoA*-1F-GC and *amoA*-2R. The optimized PCR mixture contained in a final volume of 50 μl was as follows: 2 μl of DNA (0.1 ng μl^−1^), 10 μl of 5× GoTaq Flexi buffer (Promega), 2.5 μl of MgCl_2_ (25 mM,), 1 μl of dNTPs (10 mM of each, Promega), 1 μl of each forward and reverse primers (20 μM), 0.25 μl of GoTaq Flexi polymerase (5 U μl^−1^, Promega), and 5 μl of BSA (0.1 %). PCR conditions were set as follows: 94 °C for 3 min; 17 cycles of 94 °C for 45 s, 60 °C for 45 s, followed by 72 °C for 50 s; and finally 72 °C for 10 min. The PCR product was checked by electrophoresis in 1 % agarose gel stained with GelRed (1/10,000). The PCR products were then subject to subsequent DGGE analysis.

### DGGE analysis and band sequencing

The PCR products were separated on polyacrylamide gels [6 % acrylamide–bisacrylamide (37.5:1, Bio-Rad), 1.5 mm thick, 16 × 16 cm]. The denaturant gradient ranged from 30 to 50 % denaturant [100 % denaturant was 7 M urea and 40 % formamide in 1× Tris-acetate-EDTA (TAE) buffer] for all AOPs. DGGE of the PCR products was performed with the DCode system (Bio-Rad) with 1× TAE at 60 °C, 120 V for 6 h. Gels were stained with GelRed (1.5/10,000) for 15 min and scanned with GelDoc EQ (Bio-Rad).

Two bands at the same position from two lanes were carefully excised from DGGE gels. The excised bands were washed trice by Milli-Q water, suspended in 100 μl of Milli-Q water overnight, and then re-amplified using primers without a GC clamp according to protocols described previously. The products were gel-checked and purified according to the methods described above. Each purified product was sequenced respectively by forward and reverse primers with ABI 3730xl DNA Analyzer (Applied Biosystems) at the Genome Research Centre of The University of Hong Kong.

### Phylogenetic analysis

The sequences were analyzed against those in GenBank with BLAST (Altschul et al. 1990). The sequences were aligned and phylogenetic trees were constructed using MEGA, version 5.1 (Tamura et al. 2011). For anammox bacteria, 16S rRNA gene nucleotide sequences (477 nucleotides) were used for phylogenetic analysis. For AOA and AOB, putative amino acid sequences of AmoA (198 and 150 amino acids, respectively) were used for phylogenetic analysis. Phylogenetic trees were constructed with the neighbor-joining method with 1,000 bootstrapping to estimate the confidence of tree topologies.

### Real-time quantitative PCR analysis

The abundances of anammox bacterial 16S rRNA genes and archaeal and bacterial *amoA* genes were determined in triplicate with real-time quantitative PCR amplification using a FastStart Universal SYBR Green Master (Rox) Kit (Roche, Germany). Real-time qPCR was performed in 96-well optical plates placed in the ABI PRISM® 7000 Sequence Detection System (Applied Biosystems). The primer set composed of Amx368F and Amx820R was used for the amplification of the 16S rRNA genes of anammox bacteria. The primer sets composed of Arch-*amoA*F and Arch-*amoA*R, and *amoA*-1F and *amoA*-2R were used for the amplification of the *amoA* genes of AOA and AOB, respectively. The final reaction volume was 20 μl; the reaction composition and cycling conditions were in accordance with the manual.

The specificity of the PCR amplification was determined by melting curve and gel electrophoresis. Cycle thresholds were determined by comparing with the standard curves constructed using a tenfold serial dilution (10^2^–10^7^ gene copies per microliter) of newly extracted plasmids containing corresponding gene fragments. Relative copy numbers among target groups were evaluated, and some replicates of apparent discrepancy were excluded in order to decrease standard error. The correlation coefficient, *R* ^2^, values were >0.97 for all of the standard curves.

### Nucleotide sequence accession numbers

The anammox bacterial 16S rRNA gene sequences determined in this study are available in GenBank under accession nos. JX845651–JX845660, the AOA *amoA* gene sequences under accession nos. JX845661–JX845667, and the AOB *amoA* gene sequences under accession nos. JX845668–JX845686.

## Results

### Characteristics of sediments and their pore water

Organic matter contents were variable in different types of sediments: the vegetated yellow sediments had the highest organic matter, but the non-vegetated sediments had the lowest (Table 1). In winter, the organic matter of the vegetated yellow sediments (13.0 % dry weight, DW) was higher than the vegetated black sediments (11.5 % DW) and the vegetated black sediments higher than the non-vegetated sediments (9.3 % DW). In summer, the organic matter content of the vegetated yellow sediments (11.8 % DW) was higher than the vegetated black sediments (11.0 % DW) and the vegetated black sediments higher than the non-vegetated sediments (8.5 % DW). Besides sediment type, seasonality also had an effect on the organic matter content of sediments: the same sediments always had higher organic matter content in winter than summer (Table 1).

**Table 1:** Physiochemical characteristics of sediments and pore water

Season	Sediment type	Sediment			Pore water	
		Organic matter (% DW)	pH	Redox potential (mV)	NH_4_^+^ (μM)	NO_2_^−^ (μM)
Winter	Non-VEG	9.3 ± 0.4	6.3 ± 0.7	−168.0 ± 21.5	469.9 ± 73.7	0.38 ± 0.13
	VEG-BLK	11.5 ± 0.9	6.5 ± 0.1	−73.6 ± 39.7	79.2 ± 27.8	0.25 ± 0.09
	VEG-YL	13.0 ± 1.4	6.3 ± 0.3	14.6 ± 147.8	325.3 ± 137.1	0.50 ± 0.27
Summer	Non-VEG	8.5 ± 0.3	7.3 ± 0.3	−152.4 ± 11.8	541.2 ± 60.5	0.19 ± 0.07
	VEG-BLK	11.0 ± 0.4	7.0 ± 0.1	−128.6 ± 32.8	589.4 ± 250.7	0.34 ± 0.28
	VEG-YL	11.8 ± 0.7	6.7 ± 0.3	102.0 ± 46.6	88.1 ± 8.0	0.55 ± 0.43

Each value is the mean ± SD calculated from three sediment samples of the same type in a mangrove wetland in Hong Kong

*Non*-*VEG* non-vegetated sediments, *VEG*-*BLK* vegetated black sediments, *VEG*-*YL* vegetated yellow sediments

In both seasons, the pH values of the vegetated yellow sediments were generally lower than the vegetated black and non-vegetated sediments (Table 1). In winter, the pH value of the vegetated yellow sediments (6.3) was the same as the non-vegetated sediments (6.3), but lower than the vegetated black sediments (6.5). In summer, the pH value of the vegetated yellow sediments (6.7) was lower than the vegetated black sediments (7.0) and the non-vegetated sediments (7.3). Seasonality also appeared to have an effect on the pH of the sediments: the pH values of the same sediments in summer were apparently higher than those in winter (Table 1).

In both seasons, the redox potentials of the vegetated yellow sediments were apparently higher than the vegetated black and non-vegetated sediments (Table 1). In winter, the redox potential of the vegetated yellow sediments (14.6 mV) was much higher than those of the vegetated black sediments (−73.6 mV) and non-vegetated sediments (−168.0 mV). In summer, the redox potential of the vegetated yellow sediments (102.0 mV) was also much higher than those of the vegetated black sediments (−128.6 mV) and non-vegetated sediments (−152.4 mV). Seasonality also appeared to have an effect on the redox potential of the sediments: the redox potential of the vegetated yellow sediments in summer was significantly higher than in winter (Table 1).

Generally, NH_4_^+^ in pore water of the non-vegetated sediments was higher than that of the vegetated sediments (Table 1). In winter, NH_4_^+^ in the pore water of the vegetated black sediment (79.2 μM) was much lower than those of the vegetated yellow sediments (325.3 μM) and the non-vegetated sediments (469.9 μM). However, in summer, NH_4_^+^ in the pore water of the vegetated yellow sediments (88.1 μM) was much lower than the vegetated black sediments (589.4 μM) and the non-vegetated sediments (541.2 μM). NH_4_^+^ showed slight seasonal changes (Table 1). Generally, NH_4_^+^ of pore water in winter was lower than in summer.

In both seasons, NO_2_^−^ in the pore water of the vegetated yellow sediments was apparently higher than the vegetated black sediments and the non-vegetated sediments (Table 1). In winter, NO_2_^−^ in the pore water of the yellow sediments (0.50 μM) was apparently higher than the vegetated black sediments (0.25 μM) and the non-vegetated sediments (0.38 μM). In summer, NO_2_^−^ in the pore water of the yellow sediments (0.55 μM) was also higher than the vegetated black sediments (0.34 μM) and the non-vegetated sediments (0.19 μM). However, seasonality did not show obvious changes on NO_2_^−^ between winter and summer (Table 1).

### Phylogeny and DGGE band patterns of anammox bacteria

**Fig. 1:**
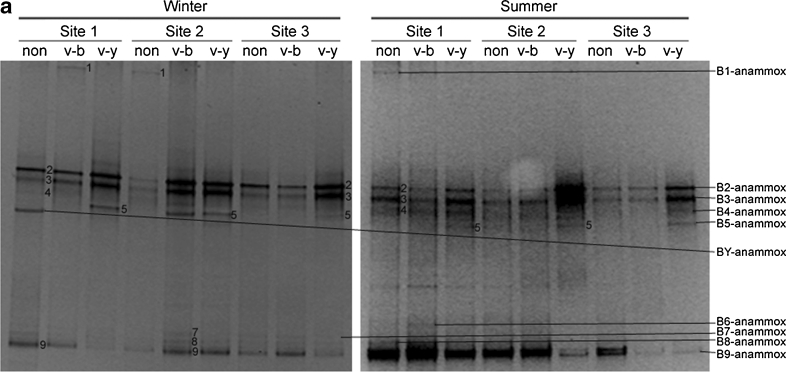

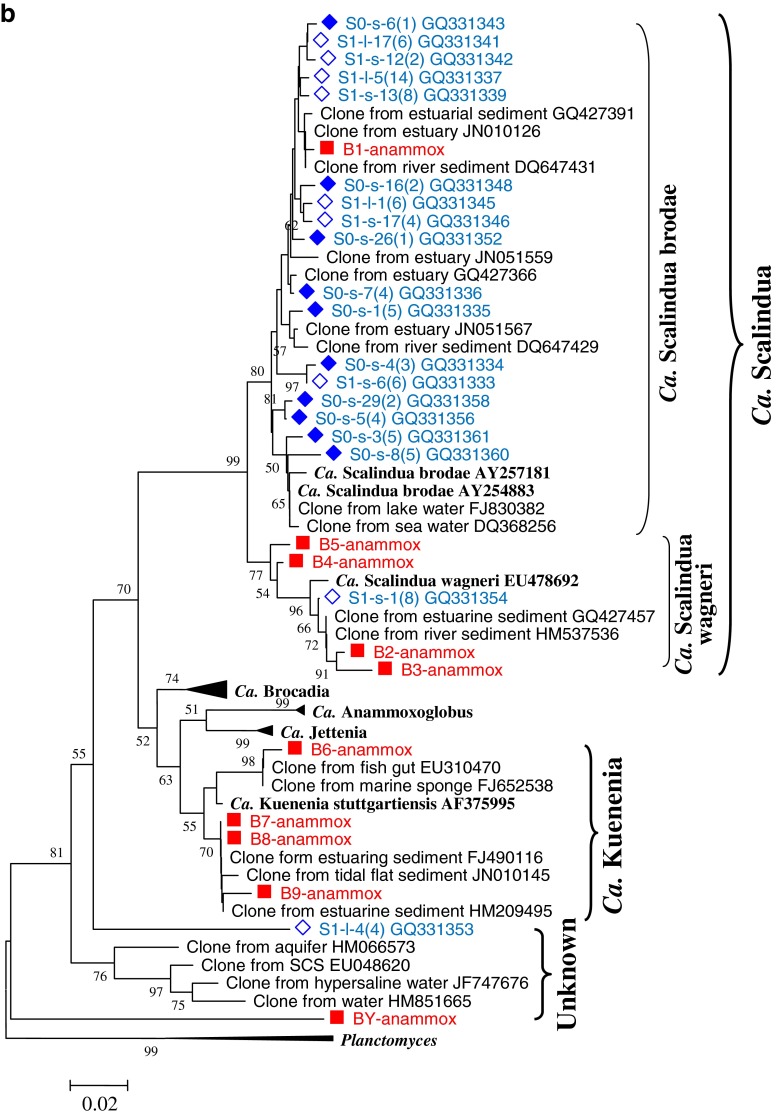
DGGE analysis (**a**) and phylogenetic tree (**b**) of anammox bacterial 16S rRNA gene fragments of different types of sediments from three sites of the mangrove wetland in winter and in summer. For DGGE, the sample ID “*non*” indicates non-vegetated sediments, “*v*-*b*” indicates vegetated black sediments, and “*v*-*y*” indicates vegetated yellow sediments. For phylogenetic tree, it was based on nucleotide sequences of 477 nucleotides. The tree was constructed with the neighbor-joining method with 1,000 bootstrapping to estimate the confidence of the tree topologies. Bootstrap values (>50 %) are indicated at the *branch points*. *Scale bar* represents 0.02 sequence divergence. *Solid squares in red* represent the DGGE band sequences of the present study. *Solid* and *hollow diamonds in blue* represent the clone sequences within and 10 m away from the vegetated area by another study in our lab

The DGGE band patterns of anammox bacteria are shown in Fig. 1a. Ten different types of bands were retrieved and sequenced, named BY-anammox and B1-anammox to B9-anammox. These bands were analyzed with MEGA 5.1; the phylogenetic tree is shown in Fig. 1b. In accordance with the phylogenetic tree, BY-anammox did not belong to any known genus of anammox bacteria, though it was affiliated to Planctomycetales. BY-anammox probably was not an anammox bacterium. Its amplification was due to the poor primer specificity of anammox species (Li et al. 2011b). Despite this, the probability of BY-anammox amplification was very low as it only occurred in 1 of the 18 samples. Excluding BY-anammox, the other nine bands could be classified into three groups. As shown in Fig. 1, the band B1-anammox, located at the upper part of the gel, belonged to *Ca*. Scalindua brodae. The bands B2-anmmox to B5-anammox, located at the middle of the gel, belonged to *Ca*. Scalindua wagneri. The bands B6-anammox to B9-anammox, located at the lower part of the gel, belonged to *Ca*. Kuenenia.

The bands B2-anammox, B3-anammox, and B9-anammox were the dominant ones in all the 18 samples (Fig. 1a). Seasonality had no observable effect on the band patterns of anammox bacteria in these samples. However, sediment type had an influence on band patterns. The band B5-anammox, related to *Ca*. Scalindua wagneri, was present in all vegetated yellow sediments. However, it was not detected in any non-vegetated sediment, and of the six vegetated black sediments, it only occurred in one sample, i.e., vegetated black sediment at site 2 in winter. B1-anammox, affiliated to *Ca*. Scalindua brodae, only existed in vegetated black and non-vegetated sediments, but not in vegetated yellow sediments. The bands B4-anammox and B6-anammox to B8-anammox were minor bands and randomly occurred in different types of sediments.

### Phylogeny and DGGE band patterns of AOA

**Fig. 2:**
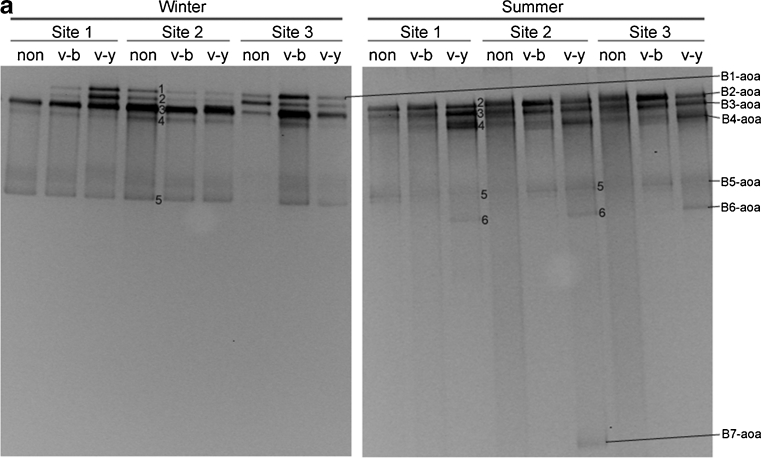

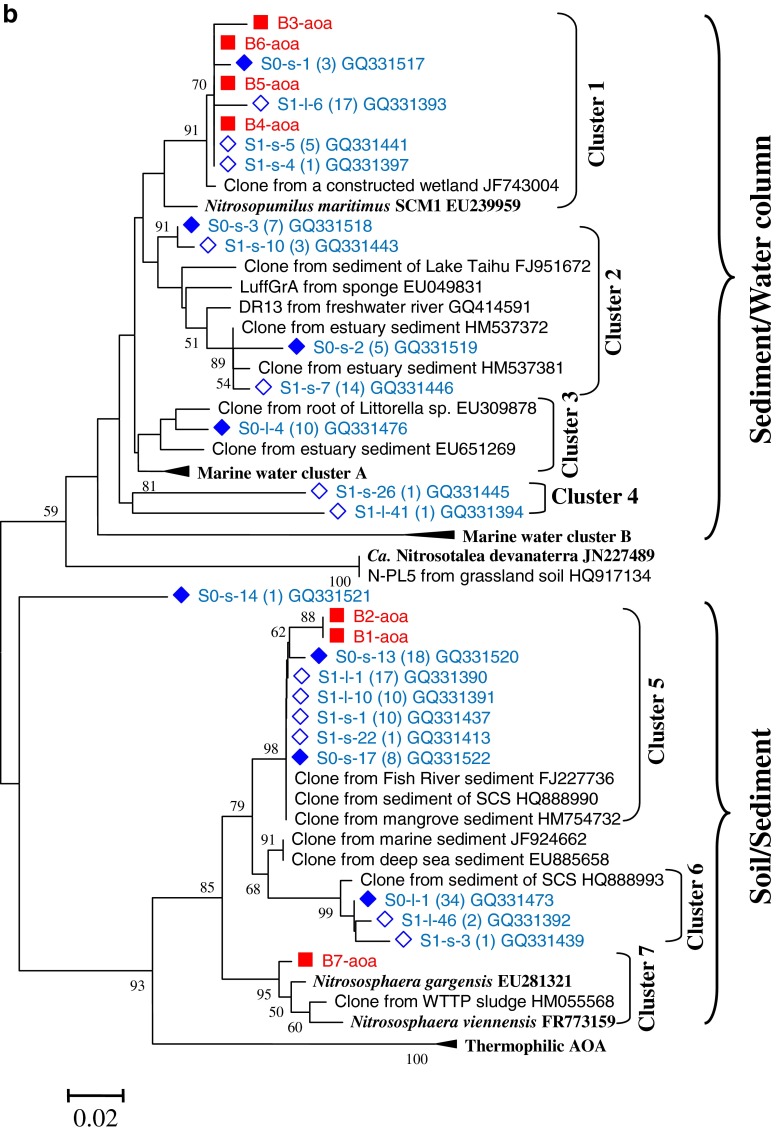
DGGE analysis (**a**) and phylogenetic tree (**b**) of AOA *amoA* genes of different types of sediments from three sites of a mangrove wetland in winter and summer. For DGGE, the sample ID “*non*” indicates non-vegetated sediments, “*v*-*b*” indicates vegetated black sediments, and “*v*-*y*” indicates vegetated yellow sediments. For the phylogenetic tree, it was based on 198 deduced amino acid sequences of the *amoA* gene sequences. The tree was constructed with the neighbor-joining method with 1,000 bootstrapping to estimate the confidence of the tree topologies. Bootstrap values (>50 %) are indicated at the *branch points*. *Scale bar* represents 0.02 sequence divergence. *Solid squares in red* represent the DGGE band sequences of the present study. *Solid* and *hollow diamonds in blue* represent the clone sequences within and 10 m away from the vegetated area by another study in our lab

The DGGE band patterns of AOA are shown in Fig. 2a. Seven different types of bands were retrieved and sequenced, named B1-aoa to B7-aoa. These bands were analyzed with MEGA 5.1; the phylogenetic tree is shown in Fig. 2b. The DGGE gel, together with the phylogenetic tree, showed that the seven types of bands could be classified into three clusters (Fig. 2). B1-aoa and B2-aoa, located at the upper part of the gel, belonged to cluster 5 within the soil/sediment clade. The bands B3-aoa to B6-aoa, located in the middle of the gel, belonged to cluster 1 within the sediment/water column clade. B7-aoa, located at the lower part of the gel, belonged to cluster 7 within the soil/sediment clade.

The bands B2-aoa to B5-aoa were dominant in all sediments (Fig. 2a). Seasonality had a noticeable effect on the AOA band patterns. The band B1-aoa was ubiquitous in all sediments of winter, but it was absent from all sediments of summer. Furthermore, the band B6-aoa did not occur in any sediment of winter, but it occurred in all vegetated yellow sediments of summer. B7-aoa was only detected in the vegetated yellow sediment of site 2 in summer. In winter, AOA band patterns were similar among different types of sediments, indicating that sediment type had no effect on AOA patterns in winter. In summer, however, the vegetated yellow sediments showed obviously different patterns from the vegetated black sediments and the non-vegetated sediments: B6-aoa existed in all vegetated yellow sediments, but not in the other two types of sediments, and B7-aoa only existed in the vegetated yellow sediment of site 2.

### Phylogeny and DGGE band patterns of AOB

**Fig. 3:**
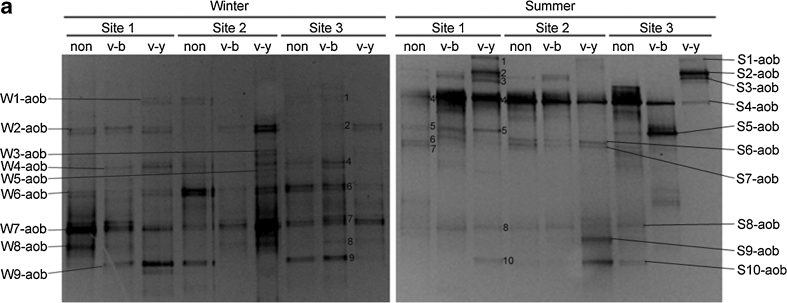

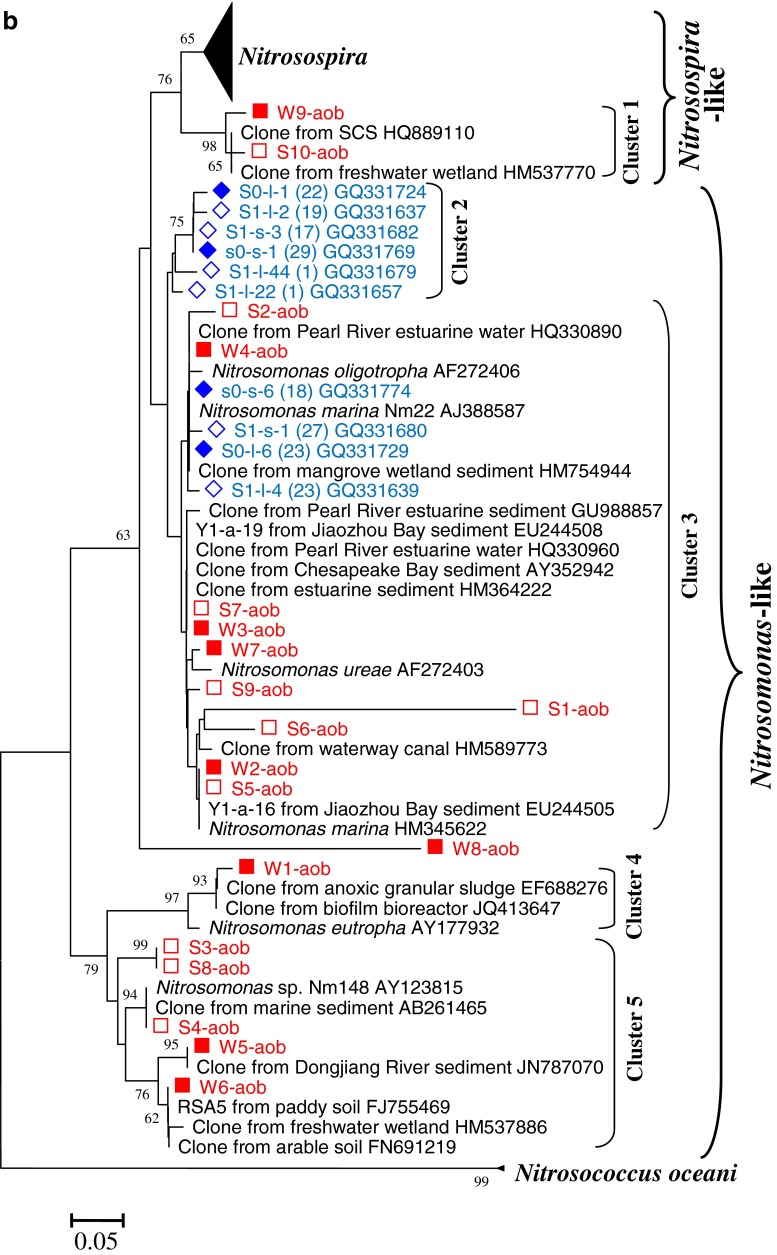
DGGE analysis (**a**) and phylogenetic tree (**b**) of AOB *amoA* genes of different types of sediments from three sites of a mangrove wetland in winter and summer. For DGGE, the sample ID “*non*” indicates non-vegetated sediments, “*v*-*b*” indicates vegetated black sediments, and “*v*-*y*” indicates vegetated yellow sediments. For the phylogenetic tree, it was based on 150 deduced amino acid sequences of the *amoA* gene sequences. The tree was constructed with the neighbor-joining method with 1,000 bootstrapping to estimate the confidence of the tree topologies. Bootstrap values (>50 %) are indicated at the *branch points*. *Scale bar* represents 0.05 sequence divergence. *Solid squares in red* represent the DGGE band sequences of the present study. *Solid* and *hollow diamonds in blue* represent the clone sequences within and 10 m away from the vegetated area by another study in our lab

The DGGE band patterns of AOB are shown in Fig. 3a. For the winter samples, many bands showed a secondary band closely next to them. The secondary bands were actually a repetitive band as their sequences were identical (data not shown). The secondary bands were produced due to the utilization of the degenerate primers. DGGE analysis could separate bands with a difference of only 1 base when the gel is well made, the denature gradient is optimum, and the running time is adequate. The gel results of winter seem to be the case. Although the repetitive bands could be avoided by not using the degenerate primers (Nicolaisen and Ramsing 2002), the results might be biased because some bands might not be amplified. If properly controlled, the DGGE results of repetitive bands will not hamper analyses because they are easily identified for the close distances. Therefore, the degenerate primers were still adopted in the present study.

Nineteen different types of AOB bands were retrieved and sequenced. Because the DGGE patterns of winter and summer were quite different, the bands of winter and summer samples were separately numbered and named: W1-aob to W9-aob for winter samples and S1-aob to S10-aob for summer samples. These sequences were analyzed with MEGA 5.1; the phylogenetic tree is shown in Fig. 3b. The phylogenetic tree showed that these bands fell into four clusters. Most of them, located at the upper part of the gel, belonged to *Nitrosomonas*-like. Only two bands, W9-aob and S10-aob, were located at the lower part of the gel, belonging to *Nitrosospira*-like. Two minor bands, W8-aob and S1-aob, were probably hybrids as no relatives could be found using the online program BLAST (Fig. 3a).

Seasonality had a significant effect on AOB band patterns (Fig. 3a). In winter, for most of the sediments, the dominant phylotypes were W2-aob, W4-aob, W6-aob, W7-aob, and W9-aob, which belong to *Nitrosomonas marina*, *Nitrosomonas oligotropha*, Cluster 5 in soils, *Nitrosomonas ureae*, and *Nitrosospira*, respectively. In summer, however, the dominant phylotypes were S4-aob and S8-aob, both related to the *Nitrosomonas eutropha*. Furthermore, although both seasons had many minor bands, few of them existed in both seasons. Compared to seasonality impact, sediment type had little effect on AOB band patterns. Though different sediments had slightly different patterns in winter, there were no obvious differences between the different types of sediments. In summer, S2-aob, S9-aob, and S10-aob seemed to be specific to the vegetated yellow sediments. S2-aob and S9-aob belonged to *Nitrosomonas*, and S10-aob probably was a member of *Nitrosospira*. Other bands did not show obvious association for any specific sediment.

### Abundances of anammox bacterial 16S rRNA genes and archaeal and bacterial *amoA* genes

**Fig. 4:**
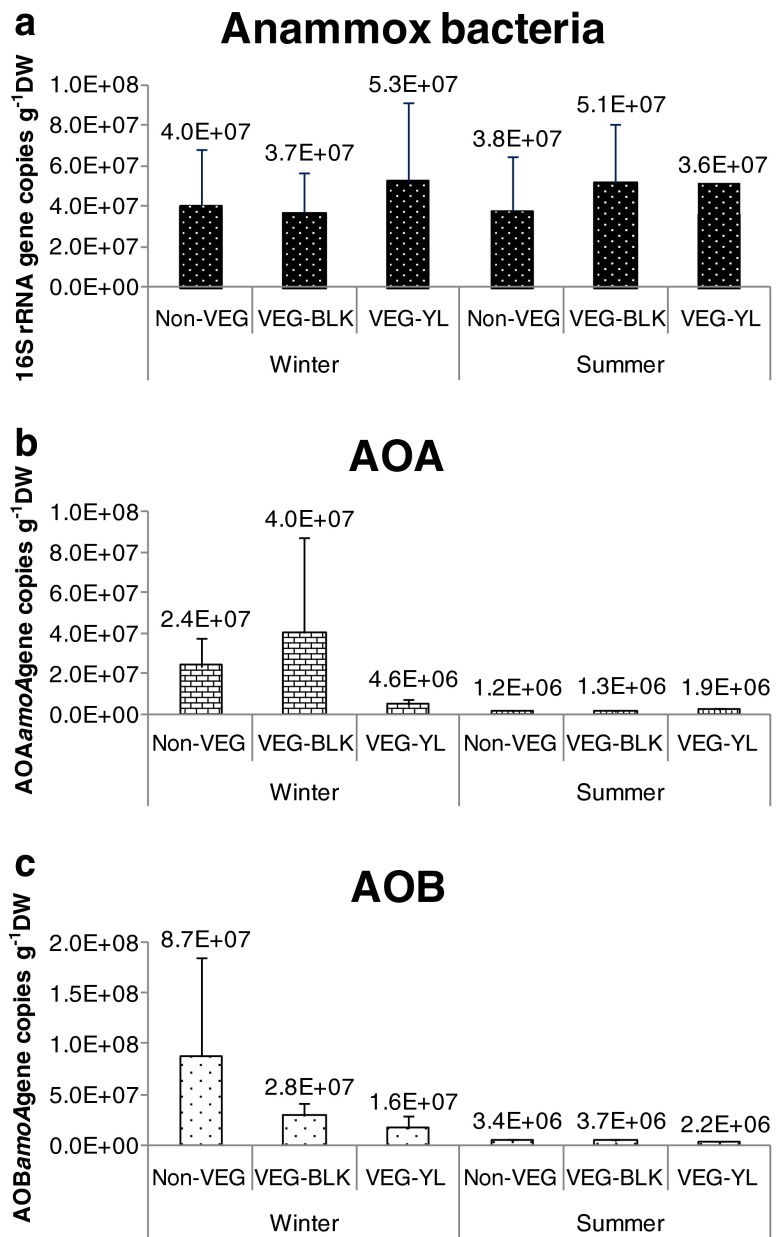
Abundances of 16S rRNA genes and archaeal and bacterial *amoA* genes in the sediments from three sites of a mangrove wetland in winter and summer. The sample ID “*Non*-*VEG*” indicates non-vegetated sediments, “*VEG*-*BLK*” indicates vegetated black sediments, and “*VEG*-*YL*” indicates vegetated yellow sediments

The abundances of anammox bacterial 16S rRNA genes in sediments did not vary significantly between winter and summer (Fig. 4a). The abundances were between 3.7 × 10^7^ and 5.3 × 10^7^ gene copies per gram DW in winter and between 3.6 × 10^7^ and 5.1 × 10^7^ gene copies per gram DW in summer. Sediment type had a small effect on the abundances of anammox bacterial 16S rRNA genes. In winter, the vegetated yellow sediments had obviously higher abundances than the other two types of sediments (Fig. 4a). In summer, the vegetated black sediments had obviously higher abundances than the other two types of sediments.

AOA abundance showed obvious seasonality. The abundances of AOA *amoA* genes in winter, ranging between 4.6 × 10^6^ and 4.0 × 10^7^ gene copies per gram DW, were significantly higher than those in summer, ranging between 1.2 × 10^6^ and 1.9 × 10^6^ gene copies per gram DW (Fig. 4b). Sediment type had an uncertain effect on the abundances of AOA *amoA* genes. In winter, archaeal *amoA* genes of the vegetated yellow sediments (4.6 × 10^6^) were apparently lower than the vegetated black sediments (4.0 × 10^7^) and the non-vegetated sediments (2.4 × 10^7^). In winter, on the contrary, the *amoA* genes of the vegetated yellow sediments (1.9 × 10^6^) were apparently higher than the vegetated black sediments (1.3 × 10^6^) and the non-vegetated sediments (1.2 × 10^6^).

Similar to AOA, AOB also showed strong seasonality. The abundances of AOB *amoA* genes in winter, ranging between 1.6 × 10^7^ and 8.7 × 10^7^ gene copies per gram DW, were apparently higher than those in summer, ranging between 2.2 × 10^6^ and 3.4 × 10^6^ gene copies per gram DW (Fig. 4c). Sediment type had an obvious effect on the abundances of AOB *amoA* genes. In winter, the *amoA* abundance of the vegetated yellow sediments (1.6 × 10^7^) was apparently lower than the vegetated black sediments (2.8 × 10^7^) and the non-vegetated sediments (8.7 × 10^7^). In summer, the *amoA* abundance of the vegetated yellow sediments (2.2 × 10^6^) was also apparently lower than the vegetated black sediments (3.7 × 10^6^) and the non-vegetated sediments (3.4 × 10^6^).

## Discussion

### The effect of sediment type and seasonality on the characteristics of sediments

Mangroves could influence the physiochemical properties of sediments by the activity of roots and leaf litters. The influences vary with the distances of the sediments from the mangrove forest. The vegetated yellow sediments are the closest to the roots of mangroves and the influence is the most intensive. The influence on the vegetated black sediments is less, and the influence on the non-vegetated sediments is the least. Because mangroves could affect sediments, seasons could also affect sediments through affecting the activity of mangroves.

In the present study, the vegetated yellow sediments had the highest organic matter contents. The vegetated yellow sediments not only received more litters than other types of sediments, roots, but also release organic substances into the rhizospheres (Lu et al. 2007), which enhanced organic matter in the vegetated yellow sediments. Organic matter of different sediments also showed a seasonal pattern: lower in summer but higher in winter. This is because organic matter could be more easily degraded by the active heterotrophic microorganisms in summer. Although a study on *K*. *obovata* showed that leaf and stipule litter exhibited clear seasonal changes, highest in July and lowest in February (Sharma et al. 2012), litters from newly fallen leaves may not enhance the organic matter within the sediments significantly.

The vegetated yellow sediments had the highest redox potentials, especially in summer, which could be the results of roots releasing the excessive oxygen into the rhizospheres and increasing the redox potentials (Nikolausz et al. 2008; Soda et al. 2007). In summer, more oxygen would be released due to the higher metabolism of plants, and this contributed to the obviously higher redox potentials of the vegetated yellow sediments in summer than in winter.

The vegetated yellow sediments had lower pH than the other types of sediments, especially in summer. This was because the pH of the rhizospheres could be changed by the activity of roots (Nye 1981). Plants could reduce pH by releasing protons when absorbing NH_4_^+^ (Ding et al. 2011). In addition, mangrove roots were demonstrated to release low-molecular-weight organic acids to decrease pH in the rhizosphere (Lu et al. 2007). This effect was more obvious in summer because of the more active physiological activities of the roots in that season.

In summer, NH_4_^+^ of the vegetated yellow sediments was apparently lower than the other two types of sediments, which was probably because of the strong absorption of the roots. In winter, interestingly, NH_4_^+^ of the vegetated black sediments was apparently lower than the other two types of sediments. Coincidently, the abundances of AOA in the vegetated black sediments in winter were apparently higher than the other two types of sediments (Fig. 4b). This suggested that the lower NH_4_^+^ was probably due to the activity of the higher quantity of AOA. Although NO_2_^−^ in the vegetated yellow sediments were higher than the other two types of sediments, NO_2_^−^ did not show apparent seasonal changes. Nitrite is the product of AOA and AOB; but at the same time the substrate of nitrite-oxidizing bacteria and thus could be easily oxidized to NO_3_^−^ (Attard et al. 2010). As a result, higher NO_2_^−^ does not implicate that there is a higher rate of ammonia oxidation.

Different types of sediments were affected by mangrove trees differently. The effect showed strong seasonality because the physiology of mangrove trees fluctuates with seasons. The differences in the characteristics of sediments will in turn affect the communities of the AOPs, which will be discussed below.

### Phylogeny of anammox bacteria and their relationship with seasonality and sediment type

The present study showed that *Ca*. Scalindua wagneri and *Ca*. Kuenenia were the dominant anammox bacteria in this wetland, and *Ca*. Scalindua brodae was a minor phylotype (Fig. 2). In contrast, Li et al. (2011c) showed that the major anammox bacteria in this wetland were *Ca*. Scalindua brodae, while *Ca*. Scalindua wagneri was only a minor phylotype; *Ca*. Kuenenia was not detected (Fig. 2). The diversity of anammox bacteria in the present study was apparently higher than those of Li et al. (2011c). One of the possible reasons was that the primer set Li et al. (2011c) used was Brod541F and Amx820R rather than Amx368F and Amx820R, which resulted in the different results here. Obviously, the primer pair of Amx368F and Amx820R used in this study could amplify broader anammox bacteria species. Another possible reason was that the samples of Li et al. (2011c) were collected from the 1- to 2-cm layer and from 20 to 21-cm layer, while ours were from 0–15 cm. Anammox bacteria were probably distributed differently by sediment depths, as Li et al. (2011c) have also shown in their study.

The vegetated yellow sediments were deeply affected by mangroves, but the vegetated black and non-vegetated sediments were less affected by mangroves. In this mangrove wetland, the dominant anammox bacteria were *Ca*. Scalindua wagneri and *Ca*. Kuenenia. *Ca*. Scalindua brodae was only a minor phylotype. Furthermore, anammox bacteria community structures had different patterns in different types of sediments (Fig. 1a). *Ca*. Scalindua wagneri was specific to vegetated yellow sediments, but *Ca*. Scalindua brodae was specific to vegetated black and non-vegetated sediments. The result suggested that *Ca*. Scalindua wagneri tended to inhabit sediments affected by mangroves while *Ca*. Scalindua brodae favored sediments not affected by mangroves. Another research showed that the dominant anammox bacteria in a young mangrove wetland in Hong Kong were *Ca*. Scalindua brodae (Wang and Gu 2012b). Because young mangroves had less effect on the sediments, the result was in agreement with our present study in that *Ca*. Scalindua brodae tended to inhabit sediments less affected by mangroves. Therefore, mangrove trees might shift anammox bacteria community structures from *Ca*. Scalindua brodae dominant to *Ca*. Scalindua wagneri and *Ca*. Kuenenia dominant in coastal wetlands.

Although community structures of anammox bacteria were related to sediment type, their abundances in different sediments did not vary much (Fig. 4a). Additionally, seasonality had no noticeable effect on either abundances or community structures of anammox bacteria. There are several explanations for this. Firstly, anammox bacteria have a very slow growth rate, and the doubling time is 11–20 days in bioreactors (Jetten et al. 2009). The slow growth rate indicates that their abundances are not likely to fluctuate greatly. Secondly, leaf litters may not have a significant effect on the growth of anammox bacteria. Although anammox bacteria were demonstrated to be mixotrophic and could utilize propionate and acetate (Güven et al. 2005; Kartal et al. 2007), in addition, genomic study showed that the freshwater species *Ca.* Kuenenia stuttgartiensis and the marine species *Ca.* Scalindua profunda are metabolic versatile and able to utilize organic matter (Strous et al. 2006; van de Vossenberg et al. 2012); they mainly utilize ammonium to acquire energy and support growth. Therefore, utilizing organic substances is probably only a supplementary pathway when ammonium or nitrite is in short supply in the environments. Furthermore, anammox bacteria could not compete for organic matter with heterotrophic microorganisms. Thus, seasonal changes of leaf litters may not affect the abundance of anammox bacteria despite them being mixotrophic. A recent study also showed that abundances of anammox bacterial 16S rRNA genes in seven wetland soils varied little between different seasons (Humbert et al. 2012), which is in agreement with our results.

### Phylogeny of AOA and their relationship with seasonality and sediment type

As shown in Fig. 2b, most AOA in the mangrove wetland were affiliated to clusters 1 and 5, which was consistent with the study of Li et al. (2011a). However, Li et al. (2011a) detected some minor phylotypes in clusters 2, 3, 4, and 6 that were not found in our study, and we detected a minor phylotype in cluster 7 which was not detected in their study. One possible reason for the difference was that AOA in this wetland were grouped by layers, as was shown in the study of Li et al. (2011a). Their sediments were from 1–2 cm and 20–21 cm layers, while ours were from a 0–15 cm layer. Another possible reason was that the PCR-DGGE approach, unlike establishing clone libraries, could not detect minor phylotypes of relatively small quantity despite of its own advantages.

Seasons had a certain effect on the community structures of AOA (Fig. 2a). The main difference of the community structures between winter and summer was that the vegetated yellow sediments in summer owned the exclusive bands B6-aoa and B7-aoa. B6-aoa was closely related to B4-aoa and B5-aoa (Fig. 2b); therefore, B6-aoa is probably genetically close to but physiologically different from B4-aoa and B5-aoa. B7-aoa was affiliated to the soil/sediment clade. B7-aoa only occurred in the vegetated yellow sediments, suggesting that the vegetated yellow sediments had the characteristics of terrestrial soils, which was the consequence of mangrove influence. This phenomenon suggested that plantation of mangroves could transform sediments of marine nature to terrestrial nature. Our investigation extended the postulation of former studies in that some phylotypes preferred marine sediments or water columns and others favored terrestrial soils (Francis et al. 2005). One thing is interesting: only in summer did the vegetated yellow sediments exhibit an effect on the community structures of AOA. This is probably because mangroves trees in summer had a stronger effect on the sediments because of their higher physiological activity.

The abundances of AOA *amoA* genes in winter were significantly higher than summer. This result is in agreement with a study on the microorganisms in the soils of a beech forest, which also showed that archaeal *amoA* genes in winter were much higher than in summer (Rasche et al. 2011). It is unknown why AOA presented such seasonal changes. AOA were demonstrated to be autotrophic (Hatzenpichler et al. 2008; Könneke et al. 2005) despite the fact that some AOA might be mixtrophic or even heterotrophic (Pester et al. 2011). As a consequence, leaf litters may not benefit autotrophic AOA. On the contrary, more leaf litters in summer may release more chemical substances such as tannin and phenolics, which probably inhibit AOA. Furthermore, in summer, the more active plants would have stronger competition for ammonia with AOA, which also could lead to lower abundances of AOA. Although seasonality had a distinctive effect on the abundance of AOA, the effect of sediment type on the abundances of AOA is uncertain as AOA in the vegetated yellow sediments were relatively lower than other sediments in winter, but higher than other sediments in summer.

### Phylogeny of AOB and their relationship with seasonality sediment type

The main phylotypes of AOB were affiliated to cluster 3 (Fig. 3b), which was in agreement with the results of Li et al. (2011a). However, we did not detect cluster 2, which was detected by Li et al. (2011a), but we detected clusters 1, 4, and 5, which Li et al. (2011a) failed to detect. Apparently, our study revealed a higher phylogenetic diversity of AOB than Li et al. (2011a) showed. The differences are presumably caused by two possibilities. For one, as Li et al. (2011a) have showed, the community structures of AOB in different layers were different. Their sediments were from 1–2 cm and 20–21 cm layers, while ours were from a 0–15 cm layer. For another, the PCR-DGGE approaches and establishing clone libraries, respectively, used in our study and theirs, may result in the differences between the two studies.

Seasonality had an apparent effect on the community structures and abundances of AOB. As described, the community structures of AOB were significantly different between winter and summer. In addition, the abundances of AOB *amoA* genes in winter were apparently higher than those in summer. This result is in agreement with a study on the microorganisms in the soils of a beech forest which also showed that bacterial *amoA* genes in winter were much higher than in summer (Rasche et al. 2011). Another study showed that AOB abundance in a New England estuary was significantly higher in spring than in late summer (Bernhard et al. 2007), which is quite similar to our results. The reason for the effect of seasonality on AOB might be the same as on AOA detailed above.

In winter, there were no obvious differences of the AOB community structures between different types of sediments. In summer, however, the AOB community structures of the vegetated yellow sediments were noticeably different from the other types of sediments. Some bands (i.e., S2-aob, S9-aob, and S10-aob) were specific to vegetated yellow sediments, which was probably because of root activity and enhancement. The abundances of AOB *amoA* genes in the vegetated yellow sediments were relatively lower than the other sediments in both seasons. This phenomenon suggested that the roots probably had a negative effect on AOB growth because roots competed for ammonia with AOB. Although roots release oxygen into the rhizosphere, which may benefit AOB, oxygen depletion in wetland sediment is a determinant of the abundance of AOB as anthropogenic pollution is very high in the sediments at the Mai Po Nature Reserve (Zhao et al. 2012a, b).

This study has shown that the community structures and abundances of AOPs in the mangrove wetland in subtropical Hong Kong showed significant seasonal fluctuations. Furthermore, sediment type had a profound effect on their community structures and abundances. The results suggested that the temporal and spatial distribution of AOPs in the mangrove wetland was dynamic. As a result, the temporal and spatial distribution of AOPs should not be neglected when studying the ecological processes of mangrove wetlands.
